# Sleep disorders in the acute phase of coronavirus disease 2019: an overview and risk factor study

**DOI:** 10.1186/s12991-023-00431-8

**Published:** 2023-01-31

**Authors:** Cun Li, Hong-bin Cai, Qing Zhou, Hua-qiu Zhang, Man Wang, Hui-cong Kang

**Affiliations:** 1 Department of Neurology, Tongji Hospital Affiliated to Tongji Medical College, Huazhong University of Science and Technology, No. 1095 Jiefang Blvd, Wuhan, 430030 People’s Republic of China; 2 Department of Neurology, Department of Pneumology, Wuhan Ninth Hospital, Wuhan, 430000 People’s Republic of China; 3 Department of Neurosurgery, Tongji Hospital Affiliated to Tongji Medical College, Huazhong University of Science and Technology, Wuhan, 430030 People’s Republic of China

**Keywords:** COVID-19, PSQI, sleep disorders, Risk factors, Carbon dioxide retention, Anemia

## Abstract

**Background:**

Sleep disorders are common during the outbreak of pandemic diseases, and similar disorders are noted in hospitalized COVID-19 patients. It is valuable to explore the clinical manifestations and risk factors for sleep disorders in COVID-19 patients.

**Methods:**

Inpatients with COVID-19 were enrolled. Detailed clinical information was collected, and sleep quality was assessed by PSQI. Patients were divided into a sleep disorder group and a normal group based on a PSQI ≥ 7, and the clinical features were compared between the groups.

**Results:**

Fifty-three patients were enrolled, and 47.2% presented sleep disorders. Sleep disorders were associated with older age (> 50), anemia and carbon dioxide retention. Furthermore, factors associated with abnormal component scores of the PSQI were: (1) patients with older age were more likely to have decreased sleep quality, prolonged sleep latency, decreased sleep efficiency, sleep disturbances, and daytime dysfunction; (2) decreased sleep quality and prolonged sleep latency were associated with dyspnea, whereas carbon dioxide retention and more lobes involved in chest CT were associated with prolonged sleep latency; (3) decreased sleep efficiency was more prevalent in patients with anemia.

**Conclusions:**

Sleep disorders were prevalent in patients during the acute phase of COVID-19, and many risk factors (older age, anemia, carbon dioxide retention, the number of lobes involved in chest CT, and dyspnea) were identified. It is important to assess the presence of sleep disorders in patients to provide early intervention.

**Supplementary Information:**

The online version contains supplementary material available at 10.1186/s12991-023-00431-8.

## Introduction

Since the outbreak of the 2019 novel coronavirus disease (COVID-19) in December 2019, 4,006,257 people have been infected, and greater than 278,000 people worldwide have died as of May 11, 2020 [1]. Therefore, COVID-19 was recognized by the World Health Organization (WHO) as a Public Health Emergency of International Concern (PHEIC) that endangers public health worldwide [2]. By binding to the receptor for angiotensin-converting enzyme 2 (ACE2), which is distributed widely in blood cells and mucous membranes [3], COVID-19 can cause multiple systemic infections and injuries, encompassing a broad spectrum from asymptomatic mild disease to severe respiratory illness along with multiple systemic organ failure and even death [4].

Mounting evidence indicates that the prevalence of novel infectious diseases, such as severe acute respiratory syndrome (SARS), could cause anxiety, depression, and stress in the general population [5], and a negative mood could influence sleep quality[6]. Similarly, one recent community study revealed that the prevalence of COVID-19 could increase sleep disorders in individuals who self-isolated for 14 days [7]. Similarly, patients with COVID-19 might suffer from sleep disorders during their disease course due to respiratory symptoms and anxiety, but information about the specific clinical characteristics and related mechanisms is limited.

COVID-19 primarily attacks the respiratory system [8], and a meta-analysis revealed that 45.6% of 656 patients presented dyspnea and hypoxemia [9], especially in the acute stage when oxygen saturation is greatly reduced and carbon dioxide retention reaches top levels. The pathophysiology is quite similar to obstructive sleep apnea syndrome (OSAS), in which hypoxemia is caused by upper airway narrowing and obstruction during sleep. OSAS leads to frequent awakenings to increase the excitability of the muscles in the airway wall to keep the airway open and increase oxygen saturation. However, frequent awakenings reduces time spent in the nonrapid eye movement (NREM) deep sleep stage and rapid eye movement (REM) sleep stage and causes sleep structure disorders and a reduction in sleep efficiency, eventually resulting in daytime lethargy and fatigue [10]. Previous studies have revealed that 48% of patients with OSAS, which is characterized by repetitive episodes of airflow cessation and brief arousal that leads to intermittent hypoxemia and hypercapnia [11], suffer from decreased sleep quality, increased sleep latency, frequent awakenings, decreased sleep efficiency and daytime dysfunction, which manifest as excessive daytime sleepiness and poor attention [10]. Given the similarity in the pathophysiology between COVID-19 and OSAS, we inferred that COVID-19 patients were highly susceptible to sleep disorders and insomnia.

Therefore, we conducted a clinical survey using the Pittsburgh Sleep Quality Index (PSQI) to investigate the sleep quality of COVID-19 patients during the acute stage of the disease. Various clinical aspects associated with the incidence of sleep dysfunction were also analyzed to illustrate the clinical risk factors for sleep dysfunction in the acute stage of COVID-19.

## Materials and methods

### Subjects

We enrolled 53 hospitalized patients identified as having nucleic acid test-confirmed COVID-19 infection in No. 9 Hospital of Wuhan City from 2 March 2020 to 11 April 2020. The mean age of our cohort was 54.26 ± 17.37 years, and the sex ratio was 1.14:1 (male: female). We defined patients over 50 years of age as the older group and those aged 50 years old or under as the younger group. Regarding education level, a low education level refers to primary school and below, a medium refers to middle school, and a high refers to university and above.

The study was approved by the Ethics Committee on Human Research of No. 9 Hospital of Wuhan City and all procedures are in accordance with the Declaration of Helsinki. All patients signed informed content to participate.

### Clinical data collection

Demographic information (age, gender, educational level, and marital status), clinical symptoms, laboratory findings, blood gas results (normal ranges are listed in Additional file 1: Tables S1 and S2), and chest computed tomographic (CT) scan findings were extracted from electronic records. Clinical symptoms consisted of infection−toxic manifestations (fever, feeble, muscle soreness, and mental fatigue), pulmonary symptoms (chest distress and dyspnea), digestive symptoms (poor appetite, emesis, stomachache, and diarrhea), and neurological symptoms (dizziness, headache, facioplegia, diplopia, hemiplegia, numbness of limbs and mood change). Laboratory findings included arterial blood gas analysis (Pouvoir Hydrogène (pH), actual bicarbonate (AB), standard bicarbonate (SB), arterial partial pressure of oxygen (PaO_2_), partial pressure of arterial carbon dioxide (PaCO_2_), total carbon dioxide (tCO_2_) and saturation oxygen (SaO_2_)), routine blood examination (leukocyte count, lymphocyte count, and hemoglobin (Hb)), blood biochemical examination (alanine transaminase (ALT), aspartate aminotransferase (AST), lactic dehydrogenase (LDH), creatine kinase (CK), coagulation function (D-dimer and fibrinogen (FIB)) and inflammation biomarker examination (hypersensitive C-reactive protein (hsCRP) and procalcitonin (PCT)). The extent of lung lesions was evaluated by the number of lobes affected as determined by chest CT, which was subdivided into six parts (left: superior lobe, ligule lobe, and inferior lobe; right: superior lobe, middle lobe, and inferior lobe).

### Sleep quality and insomnia survey using the PSQI questionnaire

The PSQI is comprised of seven components, and 18 questions are used to assess sleep quality in the month before the evaluation. Seven components include subjective sleep quality, sleep latency, sleep duration, sleep efficiency, sleep disturbances, sleep medication usage, and daytime dysfunction [12]. We calculated the total score and each component score to evaluate the specific characteristics of sleep disorders.

Consistent with a previous study [13], patients were considered to have a sleep disorder when the PSQI total score was 7 or greater and the cutoff value of the component scores of the PSQI was 1. Patients were divided into two groups: the normal group (total score < 7) and the sleep disorder group (total score ≥ 7).

### Statistical analysis

Statistical analyses were conducted using IBM SPSS Statistics Software (version 21.0, SPSS, Inc., an IBM company, Chicago, IL, USA). Continuous variables are shown as the mean ± standard deviation, and categorical variables are presented as counts (%). Independent-sample *t* tests were conducted to compare means of continuous variables. The chi-square test was used when comparing the proportions of counts between two groups, and Fisher’s exact test was applied to assess the differences in categorical variables when the sample was small (total sample number  < 40 or any expected number < 5). Significance was indicated by a *p* value  < 0.05.

## Results

### Demographic manifestations associated with sleep disorders in the acute stage of COVID-19

Of the 53 patients enrolled in our cohort, most reported a medium education level (56.6%, 30/53) followed by a high education level (35.83%, 19/53), and a low education level was the least common response (7.55%, 4/53). Regarding marital status, 43 patients (81.13%, 43/53) were married, 7 (7/53, 13.21%) were unmarried, and 3 (3/53, 5.66%) were widowed.

The mean PSQI score of the 53 patients enrolled was 7.51 ± 5.80. In total, 25 (47.2%) patients were included in the sleep disorder group, and 28 were included in the normal group based on a PSQI total score ≥ 7. The mean PSQI scores of the sleep disorder group and the normal group were 12.64 ± 4.05 and 2.93 ± 2.04, respectively. When comparing the demographic data of the normal group and sleep disorder group, several demographic factors associated with sleep disorders in COVID-19 were revealed.

Overall, the average age of patients in the sleep disorder group (PSQI total score ≥ 7) was older than that in the normal group (59.44 ± 14.69 years vs. 49.64 ± 18.80 years) (*t* test, *p* = 0.041), and age > 50 years was more common in the sleep disorder group compared with the normal group (84% (21/25) vs. 50% (14/28) (chi-square test, *p* = 0.009). In addition, regarding the components of the PSQI, older age was further indicated to be related to sleep quality and sleep latency, sleep efficiency, sleep disturbances, and daytime dysfunction in our cohort. In detail, the average age of patients with decreased sleep quality (*p* < 0.001), prolonged sleep latency (*p* = 0.043), decreased sleep efficiency (*p* = 0.037), more sleep disturbances (*p* = 0.007), and daytime dysfunction (*p* = 0.009) was greater than that noted for those without. However, no significant differences in the distribution of gender, education level, and marital status were noted between the sleep disorder and normal. Further analysis also failed to establish any correlation between all the components of the PSQI and gender, education level, or marital status in our cohort (Tables 1 and 2, and Additional file 1: Tables S3).

**Table 1:** Comparison of demographic and clinical symptoms between the normal group (PSQI total score < 7) and the sleep disorder group (PSQI total score ≥ 7)

	Total subjects (*n *= 53)	Normal group (*n* = 28)	Sleep disorders group (*n* = 25)	*p* value
Age (y, mean ± SD)	54.26 ± 17.37	49.64 ± 18.80	59.44 ± 14.69	0.041^*^
> 50	35 (66.04)	21 (75.00)	14 (56.00)	0.009^*^
≤ 50	28 (52.83)	7 (25.00)	11 (44.00)	
Gender (*n*, %)				
Male	31 (58.49)	18 (64.29)	13 (52.00)	0.365
Female	22 (41.51)	10 (35.71)	12 (48.00)	
Education level (*n*, %)				
Low	4 (7.55)	2 (7.14)	2 (8.00)	0.526
Medium	30 (56.60)	14 (50.00)	16 (64.00)	
High	19 (35.85)	12 (42.86)	7 (28.00)	
Marriage status (*n*, %)				
Unmarried	7 (13.21)	4 (14.29)	3 (12.00)	0.848
Married	43 (81.13)	22 (78.57)	21 (84.00)	
Widowed	3 (5.66)	2 (7.14)	1 (4.00)	
Clinical symptoms (n, %)				
Fever				
With	33 (62.27)	17 (60.71)	16 (64.00)	0.805
Without	20 (33.74)	11 (39.29)	9 (36.00)	
Respiratory disorders				
Chest distress				
With	28 (52.83)	12 (42.86)	16 (64.00)	0.124
Without	25 (47.17)	16 (57.14)	9 (36.00)	
Dyspnea				
With	18 (33.96)	7 (25.00)	11 (44.00)	0.145
Without	35 (66.04)	21 (75.00)	14 (56.00)	
Digestive symptoms				
With	21 (39.62)	11 (39.29)	10 (40.00)	0.958
Without	32 (60.38)	17 (60.71)	15 (60.00)	
Neurological symptoms				
With	8 (15.09)	3 (10.71)	5 (20.00)	0.577
Without	45 (84.91)	25 (89.29)	20 (80.00)	

The *p* values were obtained from independent-sample *t* tests for continuous data of age and chi-square tests and Fisher’s exact tests (total number < 40 or any expected number < 5) for categorical variables, including sex, education level, marriage status, fever, respiratory disorders, digestive symptoms and neurological symptoms

^*^p < 0.05

PSQI, Pittsburgh sleep Quality Index

**Table 2:** Age was associated with various abnormal PSQI item scores in the acute stage of COVID-19

	Total	Age (y) (mean ± SD)	*p* value	Age (y) (*n*, %)		*p* value
				> 50	≤ 50	
Sleep quality						
Decreased	38	59.46 ± 14.37	< 0.001^*^	30 (78.95)	8 (21.05)	0.002^*^
Normal	15	49.26 ± 19.06		10 (66.67)	5 (33.33)	
Sleep latency						
Prolonged	33	58.03 ± 16.32	0.043*	25 (75.76)	8 (24.24)	0.055
Normal	20	48.05 ± 18.10		10 (50.00)	10 (50.00)	
Sleep duration						
Decreased	35	57.23 ± 16.48	0.086	25 (71.43)	10 (28.57)	0.248
Normal	18	48.5 ± 18.54		10 (55.56)	8 (44.44)	
Sleep efficiency						
Decreased	24	59.75 ± 15.93	0.037*	20 (83.33)	4 (16.67)	0.016^*^
Normal	29	49.72 ± 17.76		15 (51.72)	14 (48.28)	
Sleep disturbances						
With	47	56.55 ± 16.11	0.007*	34 (72.34)	13 (27.66)	0.024^*^
Without	6	36.33 ± 19.38		1 (16.67)	5 (83.33)	
Sleep medication usage						
With	10	62.6 ± 16.18	0.095	8 (80.00)	2 (20.00)	0.506
Without	43	52.33 ± 17.44		27 (62.79)	16 (37.21)	
Daytime dysfunction						
With	31	59.48 ± 15.30	0.009*	24 (77.42)	7 (22.58)	0.038^*^
Without	22	46.91 ± 18.16		11 (50.00)	11 (50.00)	

The *p* values were obtained from independent-sample *t* tests for continuous data of age and chi-square tests and Fisher’s exact tests (total number < 40 or any expected number < 5) for categorical variable of age > 50 or not

^*^p < 0.05

PSQI, Pittsburgh sleep Quality Index

### Manifestations of sleep disorders during the acute stage of COVID-19

In our cohort, 47.2% (25/53) of patients presented sleep disorders (PSQI total score ≥ 7). For the scores of various components of the PSQI, the most common presentation of sleep disorders was sleep disturbances (88.7%, 47/53) followed by decreased sleep quality (71.7%, 38/53), prolonged sleep latency (62.3%, 33/53), shorter sleep duration (66.0%, 35/53), and daytime dysfunction (58.5%, 31/53). The least common presentations included decreased sleep efficiency (45.3%, 24/53) and sleep medication usage (18.9%, 10/53).

### Respiratory symptoms, hypoxemia, and carbon dioxide retention correlated with sleep disorders in the acute phase of COVID-19

Among subjects in our cohort, 60.4% (32/53) complained of respiratory symptoms, including chest distress or dyspnea; 52.8% (28/53) complained of chest distress, and 34.0% (18/53) presented dyspnea. All patients underwent chest CT scanning, and arterial blood gas analysis was performed in 51.0% (27/53) of the subjects. When comparing the two groups, we obtained the correlation between sleep disorders and respiratory dysfunction biomarkers.

Carbon dioxide retention (PaCO_2_ > 45 mmHg) was more commonly present in the sleep disorder group (PSQI total score ≥ 7) (5/15, 33%) than in the normal group (0/12, 0%) (Fisher’s exact test, *p* = 0.041). No significant differences in the percentage of patients with other respiratory dysfunction biomarkers, including chest distress, dyspnea, hypoxemia (PaO_2_ < 80 mmHg), the number of lobes involved in chest CT, pH value, AB, SB, tCO_2_and SaO_2_were noted between the sleep disorder group and the normal group (Tables 1 and 3, Additional file 1: Tables S1 and S2).

**Table 3:** Anemia and carbon dioxide retention were correlated with sleep disorders (PSQI total score ≥ 7) in the acute stage of COVID-19

	Total subjects	Normal group	Sleep disorders group	*p* value
Anemia (Hb < 115 g/L) (*n*, %)	53 (100)	28 (100)	25 (100)	
With	10 (18.87)	2 (7.14)	8 (32.00)	0.021^*^
Without	43 (81.13)	26 (92.86)	17 (68.00)	
Carbon dioxide retention (PaCO_2_ > 45 mmHg) (*n*, %)	27 (100)	13 (100)	14 (100)	
With	5 (18.52)	0 (0.00)	5 (35.71)	0.041^*^
Without	22 (81.48)	13 (100.00)	9 (64.29)	

The *p* values were obtained from the chi-square test, and Fisher’s exact test was used when the total number was < 40 or any expected number was < 5

^*^p < 0.05

PSQI, Pittsburgh sleep quality index; Hb, hemoglobin; PaCO_2_, partial pressure of arterial carbon dioxide

Please also note that carbon dioxide retention was also more likely to be present in patients with longer sleep latency (5/15, 33%) compared with those without (0/12, 0%) (Fisher’s exact test, *p* = 0.047), which is shown in Table 4

Through the analysis of the association between respiratory dysfunction biomarkers and seven components of the PSQI, we found that carbon dioxide retention was more likely to be present in patients with longer sleep latency (5/15, 33%) than in those without (0/12, 0%) (Fisher’s exact test, *p* = 0.047). Dyspnea was associated with impaired sleep quality and sleep latency. Thus, the percentages of patients with dyspnea among patients with decreased sleep quality (42.1% (16/38) vs. 13.3% (2/15), *p* = 0.046) and prolonged sleep latency (45.4% (15/33) vs. 15% (3/20), *p* = 0.023) were greater than that noted among those without. The number of lobes involved in chest CT was greater in subjects with prolonged sleep latency (4.19 ± 1.98) compared with those without prolonged sleep latency (2.74 ± 2.33) (*p* = 0.022). However, we did not find an association between chest distress, hypoxemia, pH value, AB, SB, tCO_2,_ or SaO_2,_ and any of the components of the PSQI (Table 4, Additional file 1: Tables S3, and S4).

**Table 4:** Respiratory dysfunction-related factors (dyspnea, anemia, greater number of lobes involved in chest CT and carbon dioxide retention (PaCO_2_ > 45 mmHg)) were associated with abnormal PSQI scores in the acute stage of COVID-19

	Total	Dyspnea (*n*, %)		*p* value	Anemia (*n*, %)		*p* value	The number of lobes involved in chest CT (mean ± SD)	*p* value	Total	Carbon dioxide retention (PaCO2 > 45 mmHg) (n, %)		*p* value
		With	Without		With	Without					With	Without	
Sleep quality													
Decreased	38	16 (42.11)	22 (57.89)	0.046*	8 (21.05)	30 (78.95)	0.797	3.92 ± 2.08	0.179	22	5 (22.73)	17 (77.27)	0.547
Normal	15	2 (13.33)	13 (86.67)		2 (13.33)	13 (87.67)		3.00 ± 2.45		5	0 (0.00)	5 (100.00)	
Sleep latency													
Prolonged	33	15 (45.45)	18 (54.55)	0.023*	9 (27.27)	24 (72.73)	0.139	4.19 ± 1.98	0.022^*^	15	5 (33.33)	10 (66.67)	0.047*
Normal	20	3 (15.00)	17 (85.00)		1 (5.00)	19 (95.00)		2.74 ± 2.33		12	0 (0.00)	12 (100.00)	
Sleep duration													
Decreased	35	14 (40.00)	21 (60.00)	0.196	8 (22.86)	27 (77.14)	0.506	3.94 ± 2.02	0.117	19	5 (26.32)	14 (73.68)	0.280
Normal	18	4 (22.22)	14 (77.78)		2 (11.11)	16 (88.89)		2.88 ± 2.47		8	0 (0.00)	8 (100.00)	
Sleep efficiency													
Decreased	24	11 (45.83)	13 (54.17)	0.097	8 (33.33)	16 (66.67)	0.036*	4.22 ± 1.98	0.095	13	4 (30.77)	9 (69.23)	0.165
Normal	29	7 (24.14)	22 (75.86)		2 (6.90)	27 (93.10)		3.18 ± 2.31		14	1 (7.14)	13 (92.86)	
Sleep disturbances													
With	47	17 (36.17)	30 (63.83)	0.623	8 (17.02)	39 (82.98)	0.684	3.80 ± 2.11	0.178	25	5 (20.00)	20 (80.00)	1.000
Without	6	1 (16.67)	5 (83.33)		2 (33.33)	4 (66.67)		2.50 ± 2.81		2	0 (0.00)	2 (100.00)	
Sleep medication usage													
With	10	3 (30.00)	7 (70.00)	1.000	3 (30.00)	7 (70.00)	0.582	3.80 ± 2.20	0.810	5	2 (40.00)	3 (60.00)	0.221
Without	43	15 (34.88)	28 (65.12)		7 (16.28)	36 (83.72)		3.61 ± 2.24		22	3 (13.64)	19 (86.36)	
Daytime dysfunction													
With	31	12 (38.71)	19 (61.29)	0.386	7 (22.58)	24 (77.42)	0.643	3.93 ± 2.02	0.296	19	5 (26.32)	14 (73.68)	0.280
Without	22	6 (27.27)	16 (72.73)		3 (13.64)	19 (86.36)		3.27 ± 2.43		8	0 (0.00)	8 (100.00)	

The *p* values were obtained from independent-sample *t* tests for continuous data of the number of lung lobes involved in chest CT and chi-square tests and Fisher’s exact tests (total number < 40 or any expected number < 5) for categorical variables, including dyspnea, anemia and carbon dioxide retention

^*^p < 0.05

PSQI, Pittsburgh sleep quality index; CT, computerized tomography; PaCO_2_, partial pressure of arterial carbon dioxide

### Anemia is associated with sleep disorders in the acute phase of COVID-19

Furthermore, we also analyzed the association between anemia and sleep disorders in our COVID-19 cohort and found that anemia was more common in the sleep disorder group (PSQI total score ≥ 7) (8/25, 32%) compared with the normal group (2/28, 8%) (chi-square test, *p* = 0.021). Moreover, anemia was more likely to be present in patients with decreased sleep efficiency (8/24, 33%) than in those without (2/29, 7%) (chi-square test, *p* = 0.036). However, we did not find any correlation between anemia and any of the other six components of the PSQI except sleep efficiency (Tables 3 and 4).

### Factors not associated with sleep disorders during the acute phase of COVID-19

In addition, we further analyzed whether immune and inflammatory factors, including fever, leukocyte count, lymphocyte count, hsCRP, and PCT, were associated with the incidence of sleep disorders in our COVID-19 cohort. However, we did not find significant differences between the sleep disorder group and the normal group for the factors above, including the percentage of patients with fever, leukocyte count, lymphocyte count, hsCRP level, and PCT level. Further analysis also did not indicate any association between these factors and the seven components of the PSQI, except for the association between PCT level and sleep efficiency (*p* = 0.047) and leukocyte count and sleep efficiency (*p* = 0.030). However, the limited patient number in our cohort prevented us from establishing any correlations (Additional file 1: Tables S1 and S4).

Besides, no significant differences in the proportion of patients with increased D-dimer, FIB, CK, or LDH levels were noted between the sleep disorder group and the normal group or between patients with or without any of the components of the PSQI (Additional file 1: Tables S1 and S4). Furthermore, no association was found between increased D-dimer or FIB and any of the components of the PSQI (Additional file 1: Tables S1 and S4).

Finally, no significant differences in the percentage of patients with digestive symptoms, neurological symptoms, elevated alanine transaminase (ALT) levels, and aspartate aminotransferase levels (AST) were noted between the sleep disorder group and the normal group or between patients with or without any of the components of the PSQI (Additional file 1: Tables S1 and S4).

## Discussion

Based on the high prevalence of anxiety, depression and sleep disorders during the COVID-19 outbreak revealed by limited literature [14], our study further explored the clinical incidence and specific features of sleep disorders evaluated by PSQI components along with the associated clinical risk factors during the acute stage of COVID-19.

As evidenced by previous SARS and Middle East respiratory syndrome (MERS) outbreaks, viral infections and subsequent quarantine can quickly culminate in sleep disorders [15]. A meta-analysis that enrolled 3559 SARS or MERS patients revealed that 41.9% experienced sleep disorders during the acute phase of their illness [16]. Similarly, 47.2% of patients in our cohort exhibited sleep disorders, which was higher than the pooled sleep disorder prevalence of 34.0% in COVID-19 inpatients reported by a Meta-analysis including 1795 subjects [17]. Compared with the incidence of sleep disorders (18.2%) in the general population [18], epidemic data from our cohort indicated that the pandemic increased the incidence of sleep disorders in COVID-19 patients, especially in hospitalized patients during the acute phase.

Our cohort revealed several risk factors associated with sleep disorders in the acute stage of COVID-19. The first important factor is older age (> 50 years), which significantly increases the incidence of sleep disorders, including decreased sleep quality, decreased sleep efficiency, prolonged sleep latency, sleep disturbances and daytime dysfunction. However, Pinto et al. showed that a relatively younger age (≤ 65 years) was a risk factor for sleep disorders along with prolonged sleep latency and sleep disturbances [19]. The contradictory results might be attributed to the following: 1) most patients in Pinto et al.’s study, who quarantined at home had relatively mild symptoms, whereas our patients were all hospitalized during the acute stage with more severe symptoms. 2) Greater than half (58%) of the patients in Pinto et al.’s study had retired and were older, and they were probably not worried about work and income. In contrast, younger patients were more prone to face employment and economic insecurity, which can contribute to sleep disorders.

However, the association between female sex, education level, and marital status, and sleep disorders were not found in our cohort, which was in contrast with previous studies. For example, Barrea et al. also showed that female sex was correlated with worsening sleep quality and sleep efficiency, whereas males more commonly presented daytime dysfunction [20, 21]. Furthermore, Gu et al. found an association between low educational level and sleep disorders in COVID-19 patients [22]. The reasons for the discordance may be due to our small sample size.

Since hypoxemia and carbon dioxide retention are the main pathophysiological mechanisms related to sleep disorders in OSAS [23] and biomarkers of respiratory system involvement, including symptoms (chest distress and dyspnea), hypoxemia and carbon dioxide retention indicated by arterial blood gas analysis and widespread pulmonary lesions in chest CT, are the main clinical presentations of COVID-19, we analyzed whether those respiratory biomarkers were associated with sleep disorders in the acute stage of COVID-19. The results showed that carbon dioxide retention, dyspnea, and a greater number of lobes involved in chest CT, were correlated with sleep disorders in our cohort, which are very similar to sleep disorders in OSAS, characterized by recurrent narrowing and closure of the upper airway, leading to intermittent hypoxemia and hypercapnia [24]. Many studies have indicated that OSAS patients with dyspnea are more prone to carbon dioxide retention, which can eventually make them wake up, making it difficult to fall asleep and reducing sleep quality [25, 26]. Interestingly, in Pinto et al.’s study, 69.6% of the COVID-19 patients had sleep disorders, and 36.7% of the whole cohort had a confirmed diagnosis of OSAS at baseline [19], which is much higher than that noted in the healthy population [27]. This finding indicates that OSAS increased the incidence of sleep disorders in COVID-19. However, the negative association between hypoxemia and sleep disorders in Mazza et al.’s study was not found by us [28]. The reasons could be that the mean PaO_2_ in our cohort (125.89 ± 73.93 mmHg) was higher than in Mazza et al.’s study (97.85 ± 1.34 mmHg).

Considering that the reduced oxygen carrying capacity of red blood cells caused by anemia can affect the function of the respiratory chain, which is involved in sleep disorders associated with OSAS [29, 30], we also analyzed the association between anemia and sleep disorders in our COVID-19 cohort, which has never been reported to the best of our knowledge. One study on iron deficiency anemia (IDA) showed that IDA contributes to the incidence of sleep disorders, independent of depression and anxiety and these effects were interpreted to result from actions on the dopamine system [31], which plays an important role in sleep regulation, including the modulation of REM sleep quality, quantity, and timing [32].

Given that tumor necrosis factor-α (TNF-α) [33], hsCRP[34], inflammatory cytokines (IL2, IL4, IL6) [35] and lipid peroxidation [36] are increased in OSAS patients, we further analyzed whether immune and inflammatory factors, including fever, leukocyte count, lymphocyte count, hsCRP and PCT, were associated with the incidence of sleep disorders in our COVID-19 cohort, and found a negative association between them. Although the immune response to COVID-19 virus induces local and systemic production of cytokines, chemokines, and other inflammatory mediators [37, 38], the correlation between inflammatory markers and sleep disorders in COVID-19 patients was not evident in our cohort or Mazza et al.’s cohort [28]. This finding is consistent with one study indicating that acute sleep disorders did not significantly alter immune cell numbers [39], while chronic sleep disorders altered the relative distribution of immune cell phenotypes in routine blood examinations [40].

In view of that recent evidence has highlighted OSAS as an independent risk factor for excessive platelet activation and arterial thrombosis [41], and abnormal coagulation indices elevated muscle and liver enzymes in COVID-19 patients were frequently observed in the acute disease stage of COVID-19 [42], we conducted the correlation analysis between coagulation function indices, muscle enzymes and liver enzymes and sleep disorders; however, we did not find an association between them. Although related studies on COVID-19 are lacking, von Känel et al. confirmed that elevated serum fibrinogen levels were more prevalent in patients with prolonged sleep latency in acute myocardial infarction [43], which was quite different from that of COVID-19. Furthermore, elevated liver enzymes were more common in patients with OSAS, but this may be attributed to the fact that the OSAS patients had a higher incidence of obesity and fatty liver, which can also cause elevated liver enzymes [44].

The current study had several limitations. First, our relatively small sample size and univariate analysis may have enlarged the association of sleep disorders with some clinical factors. Second, due to the limitation of hospitalization restrictions, we only obtained PSQI scores for sleep disorders. Third, we exclusively focused on sleep disorders in acute hospitalized patients, and further studies of it in the recovery period are currently in progress.

## Conclusions

In summary, we explored and identified risk factors for sleep disorders in the acute stage of COVID-19, including older age (> 50 years), anemia, carbon dioxide retention, dyspnea, and a greater number of lobes involved in chest CT, which were either associated with the incidence of sleep disorders or specific feature sleep disorders during the acute stage of COVID-19. The accumulation of clinical cases and further investigation of sleep disorders in patients in the long recovery disease stage will integrate our overall knowledge about sleep disorders in COVID-19.

## Supplementary Information

**Additional file 1: Table S1.** Laboratory parameters and chest CT results not associated with sleep disorders (PSQI total score ≥ 7) in the acute stage of COVID-19. **Table S2.** Blood gas analysis results not associated with sleep disorders (PSQI total score ≥ 7) in the acute stage of COVID-19. **Table S3.** Demographic and clinic symptoms not associated with subitems of PSQI in the acute stage of COVID-19. **Table S4.** Laboratory parameters (n, %) and blood gas analysis results (n, %) not associated with the subitems of PSQI in the acute stage of COVID-19.

## Data Availability

The data that support the findings of this study are available on request from the corresponding author. The data are not publicly available due to privacy or ethical restrictions.

## Abbreviations

COVID-19: 2019 Novel coronavirus disease
WHO: World Health Organization
PHEIC: Public health emergency of international concern
SARS: Severe acute respiratory syndrome
OSAS: Obstructive sleep apnea syndrome
NREM: Nonrapid eye movement
REM: Rapid eye movement
PSQI: Pittsburgh sleep quality index
CT: Computed tomographic
pH: Pouvoir Hydrogène
AB: Actual bicarbonate
SB: Standard bicarbonate
PaO_2_: Arterial partial pressure of oxygen
PaCO_2_: Partial pressure of arterial carbon dioxide
tCO_2_: Total carbon dioxide
SaO_2_: Saturation oxygen
Hb: Hemoglobin
ALT: Alanine transaminase
AST: Aspartate aminotransferase
LDH: Lactic dehydrogenase
CK: Creatine kinase
FIB: Fibrinogen
hsCRP: Hypersensitive C-reactive protein
PCT: Procalcitonin
TNF-α: Tumor necrosis factor-α
MERS: Middle East respiratory syndrome
IDA: Deficiency anemia
