# Supplementary material for: Sleep disorders in the acute phase of coronavirus disease 2019: an overview and risk factor study
Source: Ann Gen Psychiatry. 2023 Jan 31;22:3. doi: 10.1186/s12991-023-00431-8 (PMC9887242; doi:10.1186/s12991-023-00431-8)
Supplement: Supplementary file 1 — Additional file 1: Table S1. Laboratory parameters and chest CT results not associated with sleep disorders (PSQI total score ≥ 7) in the acute stage of COVID-19. Table S2. Blood gas analysis results not associated with sleep disorders (PSQI total score ≥ 7) in the acute stage of COVID-19. Table S3. Demographic and clinic symptoms not associated with subitems of PSQI in the acute stage of COVID-19. Table S4. Laboratory parameters (n, %) and blood gas analysis results (n, %) not associated with the subitems of PSQI in the acute stage of COVID-19. [file 12991_2023_431_MOESM1_ESM.docx]

Table S1 Laboratory parameters and chest CT results not associated with sleep disorders (PSQI total score ≥ 7) in the acute stage of COVID-19

|  | Total subjects (n=53) | Normal group (n=28) | Sleep disorders group (n=25) | *p* value |
| --- | --- | --- | --- | --- |
| Leukocyte count (×10^9/L) (n, %) |  |  |  |  |
| Normal (3.5-9.5) | 41 (77.36) | 19 (67.86) | 22 (88.00) | 0.147 |
| Leukocytosis (> 9.5) | 7 (13.21) | 6 (21.43) | 1 (4.00) |  |
| Leukopenia (< 3.5) | 5 (9.43) | 3 (10.71) | 2 (8.00) |  |
| Lymphocyte count (×10^9/L) (n, %) ^#^ |  |  |  |  |
| Normal (1.1-3.2) | 39 (73.58) | 21 (75.00) | 18 (72.00) | 0.805 |
| Lymphopenia (< 1.1) | 14 (26.42) | 7 (25.00) | 7 (28.00) |  |
| ALT (U/L) (n, %) |  |  |  |  |
| Normal (≤ 33) | 39 (73.58) | 20 (71.43) | 19 (76.00) | 0.706 |
| Increased (> 33) | 14 (26.42) | 8 (28.57) | 6 (24.00) |  |
| AST (U/L) (n, %) |  |  |  |  |
| Normal (≤ 32) | 43 (81.13) | 22 (78.57) | 21 (84.00) | 0.879 |
| Increased (> 32) | 10 (18.87) | 6 (21.43) | 4 (16.00) |  |
| hsCRP (mmol/L) (n, %) |  |  |  |  |
| Normal (≤ 10) | 43 (81.13) | 21 (75.00) | 22 (88.00) | 0.392 |
| Increased (> 10) | 10 (18.87) | 7 (25.00) | 3 (12.00) |  |
| FIB (g/L) (n, %) |  |  |  |  |
| Normal (2-4) | 41 (77.36) | 21 (75.00) | 20 (80.00) | 0.651 |
| Increased (> 4) | 8 (15.09) | 4 (14.29) | 4 (16.00) |  |
| Decreased (< 2) | 4 (7.55) | 3 (10.71) | 1 (4.00) |  |
| PCT (ug/L) (n, %) |  |  |  |  |
| Normal (0.02-0.05) | 6 (11.32) | 4 (14.29) | 2 (8.00) | 0.764 |
| Increased (> 0.05) | 25 (47.17) | 13 (46.43) | 12 (48.00) |  |
| Decreased (< 0.02) | 22 (41.51) | 11 (39.29) | 11 (44.00) |  |
| D-dimer (ug/L) (n, %) |  |  |  |  |
| Normal (< 0.5) | 20 (37.74) | 11 (39.29) | 9 (36.00) | 0.805 |
| Increased (≥ 0.5) | 33 (62.26) | 17 (60.71) | 16 (64.00) |  |
| CK(U/L) (n, %) |  |  |  |  |
| Normal (≤ 170) | 49 (85.71) | 24 (85.71) | 25 (100.00) | 0.149 |
| Increased (> 170) | 4 (14.29) | 4 (14.29) | 0 (0.00) |  |
| LDH (U/L) (n, %) |  |  |  |  |
| Normal (135-214) | 26 (49.06) | 12 (42.86) | 14 (56.00) | 0.617 |
| Increased (> 214) | 14 (26.42) | 8 (28.57) | 6 (24.00) |  |
| Decreased (< 135) | 13 (24.53) | 8 (28.57) | 5 (20.00) |  |
| The number of lobes involved in chest CT (mean ± SD) | 3.65±2.20 | 3.15±2.30 | 4.21±2.00 | 0.087 |

The *p* values were obtained from independent-sample t tests for continuous data of the number of lobes involved in chest CT, and chi-square tests and Fisher’s exact tests (total number <40 or any expected number < 5) for categorical variables, including Hb, leukocyte count, lymphocyte count, ALT, AST, hsCRP, PCT, D-dimer, FIB, CK and LDH.

^#^ No patient presented increased lymphocyte count (>9.5×10^9/L).

PSQI, Pittsburgh sleep Quality Index; Hb, hemoglobin; ALT, alanine transaminase; AST, aspartate aminotransferase; hsCRP, hypersensitive c-reactive protein; PCT, procalcitonin; FIB, fibrinogen; CK, creatine kinase; LDH, lactic dehydrogenase; CT, computerized tomography.

Table S2 Blood gas analysis results not associated with sleep disorders (PSQI total score ≥ 7) in the acute stage of COVID-19

|  | Total subjects (n=27) | Normal group (n=13) | Sleep disorders group (n=14) | *p* value |
| --- | --- | --- | --- | --- |
| pH value ^#^ |  |  |  |  |
| Normal (7.35-7.45) | 21 (77.78) | 9 (69.23) | 12 (85.71) | 0.385 |
| Increased (> 7.45) | 6 (22.22) | 4 (30.77) | 2 (14.29) |  |
| AB (mmol/L) |  |  |  |  |
| Normal (21-28) | 15 (55.56) | 9 (69.23) | 6 (42.86) | 0.304 |
| Increased (> 28) | 11 (40.74) | 4 (30.77) | 7 (50.00) |  |
| Decreased (< 21) | 1 (3.70) | 0 (0.00) | 1 (7.14) |  |
| SB (mmol/L) |  |  |  |  |
| Normal (21-25) | 4 (14.81) | 3 (23.08) | 1 (7.14) | 0.342 |
| Increased (> 25) | 22 (81.48) | 10 (76.92) | 12 (85.71) |  |
| Decreased (< 21) | 1 (3.70) | 0 (0.00) | 1 (7.14) |  |
| Hypoxemia (PaO_2_ < 80mmHg) |  |  |  |  |
| With | 9 (33.33) | 4 (30.77) | 5 (35.71) | 1.000 |
| Without | 18 (66.67) | 9 (69.23) | 9 (64.29) |  |
| tCO_2_ (mmol/L) |  |  |  |  |
| Normal (24-32) | 22 (81.48) | 12 (92.31) | 10 (71.43) | 0.207 |
| Increased (> 32) | 3 (11.11) | 0 (0.00) | 3 (21.43) |  |
| Decreased (< 24) | 2 (7.41) | 1 (7.69) | 1 (7.14) |  |
| SaO_2_ |  |  |  |  |
| Normal (≥ 95%) | 25 (92.59) | 13 (100.00) | 12 (85.71) | 0.481 |
| Decreased (< 95%) | 2 (7.41) | 0 (0.00) | 2 (14.29) |  |

The *p* values were obtained from chi-square tests and Fisher’s exact tests (total number <40 or any expected number < 5).

**p*< 0.05, # means no patients with increased PH level (>7.45). PSQI, Pittsburgh sleep Quality Index; pH, Pouvoir Hydrogène; AB, actual bicarbonate; SB, standard bicarbonate; PaO^2^, arterial partial pressure of oxygen; tCO_2_, total carbon dioxide; SaO_2_, arterial saturation oxygen.

Table S3 Demographic and clinic symptoms not associated with subitems of PSQI in the acute stage of COVID-19

|  | Sleep quality | | *p* value | Sleep latency | | *p* value | Sleep duration | | *p* value | Sleep efficiency | | *p* value | Sleep disturbances | | *p* value | Sleep medication usage | | *p* value | Daytime dysfunction | | *p* value |
| --- | --- | --- | --- | --- | --- | --- | --- | --- | --- | --- | --- | --- | --- | --- | --- | --- | --- | --- | --- | --- | --- |
|  | Decreased | Normal |  | Prolonged | Normal |  | Decreased | Normal |  | Decreased | Normal |  | With | Without |  | With (n=10) | Without |  | With | Without |  |
| Gender |  |  |  |  |  |  |  |  |  |  |  |  |  |  |  |  |  |  |  |  |  |
| Male | 21 (55.26) | 10 (66.67) | 0.448 | 19 (57.58) | 12 (60.00) | 0.862 | 20 (57.14) | 11 (61.11) | 1.000 | 11 (45.83) | 20 (68.93) | 0.089 | 27 (57.45) | 4 (66.67) | 1.000 | 5 (50.00) | 26 (60.47) | 0.804 | 20 (64.52) | 11 (50.00) | 0.291 |
| Female | 17 (44.74) | 5 (33.33) |  | 14 (42.42) | 8 (40.00) |  | 15 (42.86) | 7 (38.89) |  | 13 (54.17) | 9 (31.03) |  | 20 (42.55) | 2 (33.33) |  | 5 (50.00) | 17 (39.53) |  | 11 (35.48) | 11 (50.00) |  |
| Education level |  |  |  |  |  |  |  |  |  |  |  |  |  |  |  |  |  |  |  |  |  |
| Low | 4 (10.53) | 0 (0.00) | 0.155 | 3 (9.09) | 1 (5.00) | 0.242 | 2 (5.71) | 2 (11.11) | 0.691 | 2 (8.33) | 2 (6.90) | 0.323 | 4 (8.51) | 0 (0.00) | 0.229 | 2 (20.00) | 2 (4.65) | 0.203 | 4 (12.90) | 0 (0.00) | 0.142 |
| Medium | 23 (60.53) | 7 (46.67) |  | 21 (63.64) | 9 (45.00) |  | 21 (60.00) | 9 (50.00) |  | 16 (66.67) | 14 (48.28) |  | 28 (59.57) | 2 (33.33) |  | 4 (40.00) | 26 (60.47) |  | 15 (48.39) | 15 (68.18) |  |
| High | 11 (28.95) | 8 (53.33) |  | 9 (27.27) | 10 (50.00) |  | 12 (34.29) | 7 (38.89) |  | 6 (25.00) | 13 (44.83) |  | 15 (31.91) | 4 (66.67) |  | 4 (40.00) | 15 (34.88) |  | 12 (38.71) | 7 (31.82) |  |
| Marriage status |  |  |  |  |  |  |  |  |  |  |  |  |  |  |  |  |  |  |  |  |  |
| Unmarried | 3 (7.89) | 4 (26.67) | 0.122 | 4 (12.12) | 3 (15.00) | 0.376 | 4 (11.43) | 3 (16.67) | 0.867 | 2 (8.33) | 5 (17.24) | 0.504 | 5 (10.64) | 2 (33.33) | 0.268 | 1 (10.00) | 6 (13.95) | 0.776 | 3 (9.68) | 4 (18.18) | 0.652 |
| Married | 32 (84.21) | 11 (73.33) |  | 26 (78.79) | 17 (85.00) |  | 29 (82.86) | 14 (77.78) |  | 20 (83.33) | 23 (79.31) |  | 39 (82.98) | 4 (66.67) |  | 8 (80.00) | 35 (81.40) |  | 26 (83.87) | 17 (77.27) |  |
| Widowed | 3 (7.89) | 0 (0.00) |  | 3 (9.09) | 0 (0.00) |  | 2 (5.71) | 1 (5.56) |  | 2 (8.33) | 1 (3.45) |  | 3 (6.38) | 0 (0.00) |  | 1 (10.00) | 2 (4.65) |  | 2 (6.45) | 1 (4.55) |  |
| Fever |  |  |  |  |  |  |  |  |  |  |  |  |  |  |  |  |  |  |  |  |  |
| With | 26 (68.42) | 7 (46.67) | 0.141 | 20 (60.61) | 13 (65.00) | 0.749 | 21 (60.00) | 12 (66.67) | 0.635 | 14 (58.33) | 19 (65.52) | 0.591 | 30 (63.83) | 3 (50.00) | 0.833 | 4 (40.00) | 29 (67.44) | 0.211 | 22 (70.97) | 11 (50.00) | 0.121 |
| Without | 12 (31.58) | 8 (53.33) |  | 13 (39.39) | 7 (35.00) |  | 14 (40.00) | 6 (33.33) |  | 10 (41.67) | 10 (34.48) |  | 17 (36.17) | 3 (50.00) |  | 6 (60.00) | 14 (32.56) |  | 9 (29.03) | 11 (50.00) |  |
| Chest distress |  |  |  |  |  |  |  |  |  |  |  |  |  |  |  |  |  |  |  |  |  |
| With | 22 (57.89) | 6 (40.00) | 0.240 | 20 (60.61) | 8 (40.00) | 0.145 | 20 (57.14) | 8 (44.44) | 0.380 | 15 (62.50) | 13 (44.83) | 0.200 | 25 (53.19) | 3 (50.00) | 1.000 | 5 (50.00) | 23 (53.49) | 1.000 | 17 (54.84) | 11 (50.00) | 0.728 |
| Without | 16 (42.11) | 9 (60.00) |  | 13 (39.39) | 12 (60.00) |  | 15 (42.86) | 10 (55.56) |  | 9 (37.50) | 16 (55.17) |  | 22 (46.81) | 3 (50.00) |  | 5 (50.00) | 20 (46.51) |  | 14 (45.16) | 11 (50.00) |  |
| Digestive symptoms |  |  |  |  |  |  |  |  |  |  |  |  |  |  |  |  |  |  |  |  |  |
| With | 16 (42.11) | 5 (33.33) | 0.556 | 14 (42.42) | 7 (35.00) | 0.592 | 13 (37.14) | 8 (44.44) | 0.607 | 9 (37.50) | 12 (41.38) | 0.774 | 18 (38.30) | 3 (50.00) | 0.913 | 3 (30.00) | 18 (41.86) | 0.740 | 14 (45.16) | 7 (31.82) | 0.328 |
| Without | 22 (57.89) | 10 (66.67) |  | 19 (57.58) | 13 (65.00) |  | 22 (62.86) | 10 (55.56) |  | 15 (62.50) | 17 (58.62) |  | 29 (61.70) | 3 (50.00) |  | 7 (70.00) | 25 (58.14) |  | 17 (54.84) | 15 (68.18) |  |
| Neurological symptoms |  |  |  |  |  |  |  |  |  |  |  |  |  |  |  |  |  |  |  |  |  |
| With | 6 (15.79) | 2 (13.33) | 1.000 | 5 (15.15) | 3 (15.00) | 1.000 | 6 (17.14) | 2 (11.11) | 0.860 | 5 (20.83) | 3 (10.34) | 0.499 | 8 (17.02) | 0 (0.00) | 0.574 | 2 (20.00) | 6 (13.95) | 1.000 | 4 (12.90) | 4 (18.18) | 0.597 |
| Without | 32 (84.21) | 13 (86.67) |  | 28 (84.85) | 17 (85.00) |  | 29 (82.86) | 16 (88.89) |  | 19 (79.17) | 26 (89.66) |  | 39 (82.98) | 6 (100.00) |  | 8 (80.00) | 37 (86.05) |  | 27 (87.10) | 18 (81.82) |  |
| Total | 38 | 15 |  | 33 | 20 |  | 35 | 18 |  | 24 | 29 |  | 47 | 6 |  | 10 | 43 |  | 31 | 22 |  |

The *p* values were obtained from chi-square tests and Fisher’s exact tests (total number <40 or any expected number < 5).

PSQI, Pittsburgh sleep Quality Index.

Table S4 Laboratory parameters (n, %) and blood gas analysis results (n, %) not associated with the subitems of PSQI in the acute stage of COVID-19

|  | Sleep quality | | *p* value | Sleep latency | | *p* value | Sleep duration | | *p* value | Sleep efficiency | | *p* value | Sleep disturbances | | p value | Sleep medication usage | | p value | Daytime disfunction | | p value |
| --- | --- | --- | --- | --- | --- | --- | --- | --- | --- | --- | --- | --- | --- | --- | --- | --- | --- | --- | --- | --- | --- |
|  | Decreased | Normal |  | Prolonged | Normal |  | Decreased | Normal |  | Decreased | Normal |  | With | Without |  | With | Without |  | With | Without |  |
| Leukocyte count (×10^9/L) |  |  |  |  |  |  |  |  |  |  |  |  |  |  |  |  |  |  |  |  |  |
| Normal (3.5-9.5) | 30 (78.95) | 11 (73.33) | 0.862 | 27 (81.82) | 14 (70.00) | 0.507 | 29 (82.86) | 12 (66.67) | 0.339 | 22 (91.67) | 19 (65.52) | 0.030^*^ | 36 (76.60) | 5 (83.33) | 0.522 | 9 (90.00) | 32 (74.42) | 0.469 | 25 (80.65) | 16 (72.73) | 0.666 |
| Leukocytosis (> 9.5) | 3 (7.89) | 2 (13.33) |  | 3 (9.09) | 4 (20.00) |  | 3 (8.57) | 4 (22.22) |  | 0 (0.00) | 7 (24.14) |  | 7 (14.89) | 0 (0.00) |  | 1 (10.00) | 6 (13.95) |  | 4 (12.90) | 3 (13.64) |  |
| Leukopenia (< 3.5) | 5 (13.16) | 2 (13.33) |  | 3 (9.09) | 2 (10.00) |  | 3 (8.57) | 2 (11.11) |  | 2 (8.33) | 3 (10.34) |  | 4 (8.51) | 1 (16.67) |  | 0 (0.00) | 5 (11.63) |  | 2 (6.45) | 3 (13.64) |  |
| Lymphocyte count (×10^9/L) ^#^ |  |  |  |  |  |  |  |  |  |  |  |  |  |  |  |  |  |  |  |  |  |
| Normal (1.1-3.2) | 27 (71.05) | 12 (80.00) | 0.749 | 22 (66.67) | 17 (85.00) | 0.142 | 24 (68.57) | 15 (83.33) | 0.409 | 16 (66.67) | 23 (79.31) | 0.299 | 34 (72.34) | 5 (83.33) | 0.933 | 7 (70.00) | 32 (74.42) | 1.000 | 22 (70.97) | 17 (77.27) | 0.608 |
| Lymphopenia (< 1.1) | 11 (28.95) | 3 (20.00) |  | 11 (33.33) | 3 (15.00) |  | 11 (31.43) | 3 (16.67) |  | 8 (33.33) | 6 (20.69) |  | 13 (27.66) | 1 (16.67) |  | 3 (30.00) | 11 (25.58) |  | 9 (29.03) | 5 (22.73) |  |
| ALT (U/L) |  |  |  |  |  |  |  |  |  |  |  |  |  |  |  |  |  |  |  |  |  |
| Normal (≤ 33) | 29 (76.32) | 10 (66.67) | 0.710 | 25 (75.76) | 14 (70.00) | 0.645 | 24 (68.57) | 15 (83.33) | 0.409 | 15 (62.50) | 24 (82.76) | 0.096 | 34 (72.34) | 5 (83.33) | 0.933 | 9 (90.00) | 30 (69.77) | 0.363 | 22 (70.97) | 17 (77.27) | 0.608 |
| Increased (> 33) | 9 (23.68) | 5 (33.33) |  | 8 (24.24) | 6 (30.00) |  | 11 (31.43) | 3 (16.67) |  | 9 (37.50) | 5 (17.24) |  | 13 (27.66) | 1 (16.67) |  | 1 (10.00) | 13 (30.23) |  | 9 (29.03) | 5 (22.73) |  |
| AST (U/L) |  |  |  |  |  |  |  |  |  |  |  |  |  |  |  |  |  |  |  |  |  |
| Normal (≤ 32) | 29 (76.32) | 14 (93.33) | 0.300 | 27 (81.82) | 16 (80.00) | 1.000 | 28 (80.00) | 15 (83.33) | 1.000 | 18 (75.00) | 25 (86.21) | 0.493 | 37 (78.72) | 6 (100.00) | 0.484 | 10 (100.00) | 33 (76.74) | 0.213 | 23 (74.19) | 20 (90.91) | 0.239 |
| Increased (> 32) | 9 (23.68) | 1 (6.67) |  | 6 (18.18) | 4 (20.00) |  | 7 (20.00) | 3 (16.67) |  | 6 (25.00) | 4 (13.79) |  | 10 (21.28) | 0 (0.00) |  | 0 (0.00) | 10 (23.26) |  | 8 (25.81) | 2 (9.09) |  |
| hsCRP (mmol/L) |  |  |  |  |  |  |  |  |  |  |  |  |  |  |  |  |  |  |  |  |  |
| Normal (≤ 10) | 29 (76.32) | 14 (93.33) | 0.300 | 28 (84.85) | 15 (75.00) | 0.599 | 29 (82.86) | 14 (77.78) | 0.939 | 20 (83.33) | 23 (79.31) | 0.984 | 37 (78.72) | 6 (100.00) | 0.484 | 9 (90.00) | 34 (79.07) | 0.729 | 23 (74.19) | 20 (90.91) | 0.239 |
| Increased (> 10) | 9 (23.68) | 1 (6.67) |  | 5 (15.15) | 5 (25.00) |  | 6 (17.14) | 4 (22.22) |  | 4 (16.67) | 6 (20.69) |  | 10 (21.28) | 0 (0.00) |  | 1 (10.00) | 9 (20.93) |  | 8 (25.81) | 2 (9.09) |  |
| FIB (g/L) |  |  |  |  |  |  |  |  |  |  |  |  |  |  |  |  |  |  |  |  |  |
| Normal (2-4) | 29 (76.32) | 12 (80.00) | 0.959 | 28 (84.85) | 13 (65.00) | 0.226 | 27 (77.14) | 14 (77.78) | 0.910 | 19 (79.17) | 22 (75.86) | 0.686 | 35 (74.47) | 6 (100.00) | 0.372 | 8 (80.00) | 33 (76.74) | 0.566 | 21 (67.74) | 20 (90.91) | 0.128 |
| Increased (> 4) | 6 (15.79) | 2 (13.33) |  | 3 (9.09) | 5 (25.00) |  | 5 (14.29) | 3 (16.67) |  | 4 (16.67) | 4 (13.79) |  | 8 (17.02) | 0 (0.00) |  | 2 (20.00) | 6 (13.95) |  | 7 (22.58) | 1 (4.55) |  |
| Decreased (< 2) | 3 (7.89) | 1 (6.67) |  | 2 (6.06) | 2 (10.00) |  | 3 (8.57) | 1 (5.56) |  | 1 (4.17) | 3 (10.34) |  | 4 (8.51) | 0 (0.00) |  | 0 (0.00) | 4 (9.30) |  | 3 (9.68) | 1 (4.55) |  |
| PCT (ug/L) |  |  |  |  |  |  |  |  |  |  |  |  |  |  |  |  |  |  |  |  |  |
| Normal (0.02-0.05) | 5 (13.16) | 1 (6.67) | 0.748 | 3 (9.09) | 3 (15.00) | 0.682 | 3 (8.57) | 3 (16.67) | 0.322 | 0 (0.00) | 6 (20.69) | 0.047^*^ | 5 (10.64) | 1 (16.67) | 0.864 | 1 (10.00) | 5 (11.63) | 0.977 | 3 (9.68) | 3 (13.64) | 0.729 |
| Increased (> 0.05) | 17 (44.74) | 8 (53.33) |  | 15 (45.45) | 10 (50.00) |  | 19 (54.29) | 6 (33.33) |  | 14 (58.33) | 11 (37.93) |  | 22 (46.81) | 3 (50.00) |  | 5 (50.00) | 20 (46.51) |  | 16 (51.61) | 9 (40.91) |  |
| Decreased (< 0.02) | 16 (42.11) | 6 (40.00) |  | 15 (45.45) | 7 (35.00) |  | 13 (37.14) | 9 (50.00) |  | 10 (41.67) | 12 (41.38) |  | 20 (42.55) | 2 (33.33) |  | 4 (40.00) | 18 (41.86) |  | 12 (38.71) | 10 (45.45) |  |
| D-dimer(ug/L) |  |  |  |  |  |  |  |  |  |  |  |  |  |  |  |  |  |  |  |  |  |
| Normal (< 0.5) | 13 (34.21) | 7 (46.67) | 0.399 | 13 (39.39) | 7 (35.00) | 0.749 | 12 (34.29) | 8 (44.44) | 0.470 | 7 (29.17) | 13 (44.83) | 0.242 | 17 (36.17) | 3 (50.00) | 0.833 | 3 (30.00) | 17 (39.53) | 0.843 | 10 (32.26) | 10 (45.45) | 0.329 |
| Increased (≥ 0.5) | 25 (65.79) | 8 (53.33) |  | 20 (60.61) | 13 (65.00) |  | 23 (65.71) | 10 (55.56) |  | 17 (70.83) | 16 (55.17) |  | 30 (63.83) | 3 (50.00) |  | 7 (70.00) | 26 (60.47) |  | 21 (67.74) | 12 (54.55) |  |
| CK(U/L) |  |  |  |  |  |  |  |  |  |  |  |  |  |  |  |  |  |  |  |  |  |
| Normal (≤170) | 34 (89.47) | 15 (100.00) | 0.466 | 32 (96.97) | 17 (85.00) | 0.288 | 33 (94.29) | 16 (88.89) | 0.877 | 23 (95.83) | 26 (89.66) | 0.299 | 43 (91.49) | 6 (100.00) | 1.000 | 10 (100.00) | 39 (90.70) | 1.000 | 30 (96.77) | 19 (86.36) | 0.376 |
| Increased (> 170) | 4 (10.53) | 0 (0.00) |  | 1 (3.03) | 3 (15.00) |  | 2 (5.71) | 2 (11.11) |  | 1 (4.17) | 3 (10.34) |  | 4 (8.51) | 0 (0.00) |  | 0 (0.00) | 4 (9.30) |  | 1 (3.23) | 3 (13.64) |  |
| LDH (U/L) |  |  |  |  |  |  |  |  |  |  |  |  |  |  |  |  |  |  |  |  |  |
| Normal (135-214) | 17 (44.74) | 9 (60.00) | 0.119 | 17 (51.52) | 9 (45.00) | 0.360 | 17 (48.57) | 9 (50.00) | 0.397 | 14 (58.33) | 12 (41.38) | 0.385 | 24 (51.06) | 2 (33.33) | 0.305 | 7 (70.00) | 19 (44.19) | 0.308 | 13 (41.94) | 13 (59.09) | 0.409 |
| Increased (> 214) | 13 (34.21) | 1 (6.67) |  | 10 (30.30) | 4 (20.00) |  | 11 (31.43) | 3 (16.67) |  | 6 (25.00) | 8 (27.59) |  | 13 (27.66) | 1 (16.67) |  | 2 (20.00) | 12 (27.91) |  | 10 (32.26) | 4 (18.18) |  |
| Decreased (< 135) | 8 (21.05) | 5 (33.33) |  | 6 (18.18) | 7 (35.00) |  | 7 (20.00) | 6 (33.33) |  | 4 (16.67) | 9 (31.03) |  | 10 (21.28) | 3 (50.00) |  | 1 (10.00) | 12 (27.91) |  | 8 (25.81) | 5 (22.73) |  |
| Total | 38 | 15 |  | 33 | 20 |  | 35 | 18 |  | 24 | 29 |  | 47 | 6 |  | 10 | 43 |  | 31 | 22 |  |
| pH value ^#^ |  |  |  |  |  |  |  |  |  |  |  |  |  |  |  |  |  |  |  |  |  |
| Normal (7.35-7.45) | 17 (77.27) | 4 (80.00) | 1.000 | 12 (80.00) | 9 (75.00) | 1.000 | 15 (78.95) | 6 (75.00) | 1.000 | 11 (84.62) | 10 (71.43) | 0.648 | 19 (76.00) | 2 (100.00) | 1.000 | 4 (80.00) | 17 (77.27) | 1.000 | 14 (73.68) | 7 (87.50) | 0.633 |
|  |  |  |  |  |  |  |  |  |  |  |  |  |  |  |  |  |  |  |  |  |  |
| Decreased (< 7.35) | 5 (22.73) | 1 (20.00) |  | 3 (20.00) | 3 (25.00) |  | 4 (21.05) | 2 (25.00) |  | 2 (15.38) | 4 (28.57) |  | 6 (24.00) | 0 (0.00) |  | 1 (20.00) | 5 (22.73) |  | 5 (26.32) | 1 (12.50) |  |
| AB (mmol/L) |  |  |  |  |  |  |  |  |  |  |  |  |  |  |  |  |  |  |  |  |  |
| Normal (21-28) | 11 (50.00) | 4 (80.00) | 0.464 | 8 (53.33) | 7 (58.33) | 0.456 | 9 (47.37) | 6 (75.00) | 0.389 | 6 (46.15) | 9 (64.29) | 0.437 | 13 (52.00) | 2 (100.00) | 0.421 | 2 (40.00) | 13 (59.09) | 0.591 | 9 (47.37) | 6 (75.00) | 0.389 |
| Increased (> 28) | 10 (45.45) | 1 (20.00) |  | 7 (46.67) | 4 (33.33) |  | 9 (47.37) | 2 (25.00) |  | 6 (46.15) | 5 (35.71) |  | 11 (44.00) | 0 (0.00) |  | 3 (60.00) | 8 (36.36) |  | 9 (47.37) | 2 (25.00) |  |
| Decreased (< 21) | 1 (4.55) | 0 (0.00) |  | 0 (0.00) | 1 (8.33) |  | 1 (5.26) | 0 (0.00) |  | 1 (7.69) | 0 (0.00) |  | 1 (4.00) | 0 (0.00) |  | 0 (0.00) | 1 (4.55) |  | 1 (5.26) | 0 (0.00) |  |
| SB (mmol/L) |  |  |  |  |  |  |  |  |  |  |  |  |  |  |  |  |  |  |  |  |  |
| Normal (21-25) | 3 (13.64) | 1 (20.00) | 0.844 | 2 (13.33) | 2 (16.67) | 0.494 | 2 (10.53) | 2 (25.00) | 0.528 | 1 (7.69) | 3 (21.43) | 0.374 | 4 (16.00) | 0 (0.00) | 0.782 | 1 (20.00) | 3 (13.64) | 0.844 | 1 (5.26) | 3 (37.50) | 0.087 |
| Increased (> 25) | 18 (81.82) | 4 (80.00) |  | 13 (86.67) | 9 (75.00) |  | 16 (84.21) | 6 (75.00) |  | 11 (84.62) | 11 (78.57) |  | 20 (80.00) | 2 (100.00) |  | 4 (80.00) | 18 (81.82) |  | 17 (89.47) | 5 (62.50) |  |
| Decreased (< 21) | 1 (4.55) | 0 (0.00) |  | 0 (0.00) | 1 (8.33) |  | 1 (5.26) | 0 (0.00) |  | 1 (7.69) | 0 (0.00) |  | 1 (4.00) | 0 (0.00) |  | 0 (0.00) | 1 (4.55) |  | 1 (5.26) | 0 (0.00) |  |
| Hypoxemia (PaO_2_<  80mmHg) |  |  |  |  |  |  |  |  |  |  |  |  |  |  |  |  |  |  |  |  |  |
| With | 9 (40.91) | 0 (0.00) | 0.136 | 6 (40.00) | 3 (25.00) | 0.683 | 6 (31.58) | 3 (37.50) | 1.000 | 4 (30.77) | 5 (35.71) | 1.000 | 9 (36.00) | 0 (0.00) | 0.538 | 1 (20.00) | 8 (36.36) | 0.636 | 6 (31.58) | 3 (37.50) | 1.000 |
| Without | 13 (59.09) | 5 (100.00) |  | 9 (60.00) | 9 (75.00) |  | 13 (68.42) | 5 (62.50) |  | 9 (69.23) | 9 (64.29) |  | 16 (64.00) | 2 (100.00) |  | 4 (80.00) | 14 (63.64) |  | 13 (68.42) | 5 (62.50) |  |
| tCO_2_ (mmol/L) |  |  |  |  |  |  |  |  |  |  |  |  |  |  |  |  |  |  |  |  |  |
| Normal (24-32) | 17 (77.27) | 5 (100.00) | 0.498 | 12 (80.00) | 10 (83.33) | 0.086 | 15 (78.95) | 7 (87.5) | 0.425 | 10 (76.92) | 12 (85.71) | 0.787 | 20 (80.00) | 2 (100.00) | 0.782 | 4 (80.00) | 18 (81.82) | 0.640 | 15 (78.95) | 7 (87.50) | 0.425 |
| Increased (> 32) | 3 (13.64) | 0 (0.00) |  | 3 (20.00) | 0 (0.00) |  | 3 (15.79) | 0 (0.00) |  | 2 (15.38) | 1 (7.14) |  | 3 (12.00) | 0 (0.00) |  | 1 (20.00) | 2 (9.09) |  | 3 (15.79) | 0 (0.00) |  |
| Decreased (< 24) | 2 (9.09) | 0 (0.00) |  | 0 (0.00) | 2 (16.67) |  | 1 (5.26) | 1 (12.5) |  | 1 (7.69) | 1 (7.14) |  | 2 (8.00) | 0 (0.00) |  | 0 (0.00) | 2 (9.09) |  | 1 (5.26) | 1 (12.50) |  |
| SaO_2_ |  |  |  |  |  |  |  |  |  |  |  |  |  |  |  |  |  |  |  |  |  |
| Normal (≥ 95%) | 20 (90.91) | 5 (100.00) | 1.000 | 9 (60.00) | 9 (75.00) | 0.683 | 13 (68.42) | 5 (62.5) | 1.000 | 9 (69.23) | 9 (64.29) | 1.000 | 16 (64.00) | 2 (100.00) | 0.538 | 4 (80.00) | 14 (63.64) | 0.636 | 13 (68.42) | 5 (62.50) | 1.000 |
| Decreased (< 95%) | 2 (9.09) | 0 (0.00) |  | 6 (40.00) | 3 (25.00) |  | 6 (31.58) | 3 (37.5) |  | 4 (30.77) | 5 (35.71) |  | 9 (36.00) | 0 (0.00) |  | 1 (20.00) | 8 (36.36) |  | 6 (31.58) | 3 (37.50) |  |
| Total | 22 | 5 |  | 15 | 12 |  | 19 | 8 |  | 13 | 14 |  | 25 | 2 |  | 5 | 22 |  | 19 | 8 |  |

The *p* values were obtained from chi-square tests and Fisher’s exact tests were used when total number < 40 or any expected number < 5.

**p*< 0.05, ^#^ no patient with lymphocyte count > 3.2×10^9/L and increased PH level (> 7.45).

PSQI, Pittsburgh sleep Quality Index; ALT, alanine transaminase; AST, aspartate aminotransferase; hsCRP, hypersensitive c-reactive protein; PCT, procalcitonin; FIB, fibrinogen; CK, creatine kinase; LDH, lactic dehydrogenase, pH, Pouvoir Hydrogène; AB, actual bicarbonate; SB, standard bicarbonate; PaO_2_, arterial partial pressure of oxygen; tCO_2_, total carbon dioxide; SaO_2_, arterial saturation oxygen.
